# Christian Science “The Religo-Medical Masquerade”

**Published:** 1910-10

**Authors:** Henry R. Slack

**Affiliations:** Superintendent La Grange Sanatorium, La Grange, Ga.


### CHRISTIAN SCIENCE "THE RELIGIO-MEDICAL MASQUERADE."

BY HENRY R. SLACK, PH. M., M. D.

SUPERINTENDENT LA GRANGE SANATORIUM, LA GRANGE, GA.

In his address before the Sanhedrim Gamaliel, the learned doctor of the law cited numerous examples of imposters leading many men astray, and among them "Thaddeus boasting himself to be somebody, to whom a number joined themselves, and all, as many as obey him, were scattered and brought to naught."

As it was then, so it is now, and, I fear, ever shall be, many are always ready to be "led astray by any wind of doctrine." The latest and most successful of these false teachers is Mrs. Mary Baker Glover Patterson Eddy, the founder of Christian Science, a misnomer, for it is neither Christian nor Science, but as Peabody calls it, a "Religio-Medical Masquerade."

For the facts I shall present to you I am indebted to Dr. Harry T. Marshall, professor of pathology in the University of Virginia, who made a study of this cult when a felolw in patholl-ogy in the Johns Hopkins University, and to Mr. Fredrick W. Peabody, LL. B., of the Boston Bar, who probably has had better opportunity to study the teachings of Mrs. Eddy and herself than any one who has undertaken the task. I shall quote liberally from both.

It is always interesting and instructive to study the biographies of men and women who have done something. Especially in this true in cases of the founders of new religions, for "Ye cannot gather figs of thistles" and "By their fruits ye shall know them."

Mary Baker Glover Eddy was born in the town of Bow, N. H., July 16, 1821, of good New England parentage, but received only a very limited education. She claimed to have studied the classics and even to have knowledge of Hebrew, but Peabody, who has had to read many of her unedited writings, says "She has never been on any but the most distant speaking terms with her mother tongue." At the age of seventy in writing she attributes the authorship of Dickens' Pickwick Papers to Washington Irving. The first fifty years of her life were spent in extreme poverty, and at one time she was a Spiritualist medium about Boston.

In December, 1843, she married Geo. W. Glover, a young brick layer by trade and with him moved to Wilmington, N. C., where he shortly afterwards died of yellow fever. Her only son was born six months after the death of her husband and was unfeelingly abandoned by her in infancy and reared in a Western State in ignorance. In 1853 she married Daniel Patterson, an itinerant dentist, but their pathway was not strewn with flowers; and after twenty years of connubial infelicity she was granted a divorce on the grounds of desertion.

Mrs. Eddy does not believe in marriage for others, but she contracted a third alliance herself with Gilbert A. Eddy, January 1, 1877. In the record of this marriage the bride's age is given as forty, instead of fifty-six, which was her true age. This marriage was not ideal, as Mr. Eddy complained that neither he nor God Almighty could please his exacting spouse. Death released him from his bondage in 1882, despite his miracle working wife's having twice brought him back to life. Many believe she has contracted a fourth marriage with one Calvin A. Frye.

And this is the woman who claims to be referred to by prophecy of St. John in Revelations, twelfth chapter, "And there appeared a great wonder in Heaven, a woman clothed with the sun and the moon under her feet, and upon her head a crown of twelve stars." In the earlier days she placed her mission above that of Jesus, inasmuch as the idea of God given her was, she said, "higher, clearer and more permanent than before," but later she is satisfied with equality. She points out the short comings of Jesus thus, "Our Master heaeld the sick, practiced Christian healing, and taught generalitics of this divine principle to his students, but left no definite word for demonstrating his principles of healing and preventing disease." "This remained to be discovered by Christian Science. Had wisdom characterized all of his sayings, he would not have prophesied his own death and thereby hastened or caused it." While in speaking of herself she said, "The works I have written on Christian Science contain absolute truth and my necessity was to tell it. I was a scribe under orders, and who can refrain from transcribing what God indites?" So wisdom, according to Mrs. Eddy, did not characterize all the sayings of Jesus, but she speaks absolute truth.

Thus she would lead one to believe that "Science and Health with the Key to the Scriptures" is a divine inspiration, an absolute truth. Here are some of its truths:

"The less you know or think about Hygiene, the less we are predisposed to sickness. Treatises on anatomy, physiology and health sustained by material law are promoters of sickness and disease. It is proverbial that as long as you read medical works you will be sick."

According to this truth physicians should be prone upon beds of languishing most of the time, and the presence of so many healthy looking specimens of the Esculapian art here I hold proves this to be a lie, "mortal mind."

Again, "the blood, brain and lungs, etc., have nothing to do with life." "Gender is also a quality, a characteristic of mind and not of mother."

"The daily ablutions of an infant are no more natural or necessary than would be the process of taking a fish out of water every day and covering it with dirt, in order to make it thrive more vigorously thereafter in its native elements."

This "rot" inspired of God! Mark Twain says, "I cannot see how any one contemplating Mrs. Eddy's career can deny to the Divine Being the possession of a sense of humor. God is so amused by Mrs. Eddy's accomplishments that he is prompted to laughter, and Christian Science thus excapes the consuming fire of Divine wrath.

The absurdity of Divine inspiration is shown by the followng: James Henry Wiggin, an ex-Unitarian minister was for years Mrs. Eddy's literary expert, putting al her productions; including the inspired book, into good English and as coherent form as she would permit. He wrote a sermon for Mrs. Eddy to preach, which she preached as her own, and subsequently incorporated, with some slight additions, which really named Mr. Wiggin's work in her God-inspired book as a chapter entitled "Wayside Hints." This chapter is left out of the last additions, but it was given to thtet ttwotrld with the rest in the thirty-sixth edition as of God's authorship. Many times has Mr. Wiggin been heard to say with a chuckle of amusement that it was a source of much mirth to him to hear from time to time Mrs. Eddy's devotees exclaim, with pious earnestness, that the chapter he had written at so much per word, was the most divinely inspired portion of the divine volume.

In October, 1862, Mrs. Mary Patterson, now Mrs. Eddy, placed herself under the professional care of Dr. P. P. Quimby, of Portland, Me., a magnetic healer, with result that within a week's time she was able to climb 182 steps to the dome of the city hall. "This truth which he opposes to error of giving intelligence to matter and placing pain where it never placed itself, if received understandingly, changes the current of the system to their normal action and the mechanism of the body goes on undisturbed." "This is the Key to Christian Science healing, i. e., suggestive therapeutics which she appropriated from old Dr. Quimby, but did not begin to practice until after his death in 1866. At his death she wrote some verses "to Dr. P. P. Quimby, who Healed the sick as Jesus did." Now she speaks slightingly of the old Doctor's mesmeric treatment giving her only temporary relief.

Her description of the discovery is thus described: "In company with her husband she was returning from an errand of mercy, when she fell upon the icy curbstone and was carried helpless to her home. The skilled physicians declared that there was no hope for her, and pronounced the verdict that she had but three days to live. Finding no hope and no help on earth, she lifted her heart to God. On the third day, calling for her Bible, she asked the family to leave the room. Her Bible opened at the healing of the palsied man, Matthew IX, 2."

The truth which set him free she saw.
The power which gave him strength she felt.

And life Divine which healed the sick of the palsy restored her, and she rose from the bed of pain, healed and free. When she walked into the midst of the family they cried out in alarm, thinking that she had died and that they beheld her ghost. This miraculous restoration dates the birth of Christian Science."

This is a touching and moving story, but it is only a story, for the physcian who attended her at that time is still living an honored member of the professon and is practicing in Springfield, Mass. Dr. Alvin M. Cushing under oath says that he did not considered Mrs. Patterson in a critical condition or that there was no hope for her, or that she had but three days, or any other limited number of days to live. That she did not pretend to be miraculously cured on the third or any other day of her illness; that he gave her medicine on that day and later days in her illness, and was called to see her again four or five times in August of the same year and gave her medicine. Dr. Cushing is positive because he belongs to that accurate class of physicians who keep a record book of all cases, and he still has the record of Mrs. Patterson's (Eddy's) case.

The founders of new religions must, of necessity, be able to work miracles in order to impress their disciples with their Divine power. Mrs. Eddy is not in the least backward in claiming this power. In a signed article in the New York Sun, December 16, 1898, she says, "I challenge the world to disprove what I hereby declare. After my discovery of Christian Science I healed consumption in its last stages, that the M. D.'s by verdict of the stethoscope and the schools declared incurable, the lungs eing mostly consumed. I healed malignant tubercular diphtheria and carious bones that could be dented by the finger, saving them when the surgeon's instruments were lying on the table ready for their amputation. I have healed at one visit a cancer that had eaten the flesh of the neck so as to expose the jugular vein, so that it stood out like a cord."

Dr. C. A. L. Reed, Ex-President, of the A. M. A., challenged Mrs. Eddy in the issue of the Sun, January 1, 1899, to prove her miraculous cures. He offered to furnish cases identical with those she claimed to have cured and said that if she would heal any one of them, he would proclaim her omnipotence from the house tops; and, if she would cure all or half of them, he would cheerfully crawl on his knees that he might but touch the hem of her garment.

The following directions are taken from the Christian Science text book and illustrate the Christian Science method of practice.

(Pages 410-411.)

"Always begin your treatment by allaying the fear of your patient. Silently reassure the patient as to his exemption from disease and danger. Watch the result of this simple rule of Christian Science, and you will find that it alleviates the symptoms of every disease. If you succeed in wholly removing the fear, your patient is healed. The great fact that God wisely governs all, never punishing aught but sin, is your standpoint whence to advance and destroy the human fear of sickness. Plead the case in Science and for truth, mentally and silently. You may vary the arguments to meet the peculiar or general symptoms of the case you treat; but be thoroughly persuaded in your own mind and you will finally be winner."

"You may call the disease by name when you mentally deny it but by naming it audibly you are liable to impress it upon the thought. Argue with the patient (mentally, not audibly) that he has no disease. If the case is in a young child or an infant, it needs to be met mainly through the parents, thoughts silently or audible, on the basis of Christian Science."

(Pages 422-23). "If the case to betreated is consumption, take up the leading points included, according to belief, in this disease. Show that it is not inherited; that inflammation, tubercles, hemorrhage and decomposition are beliefs, images of moral thought, superimposed upon the body; that they are not the truth of men; that they should be treated as error and put out of thought. Then these ills will disappear. If the lungs are disappearing, this is but one of the beliefs of mortal mind. Mortal man will be less mortal when he learns that lungs never sustained existence, and can never destroy God, who is our life. What if the lungs are ulcerated? God is more to man than his lungs, and the less we acknowledge matter of its laws the more immortality we possess."

Christian Science is the greatest "get-rich-quick" religion ever invented, and does not appeal or apply to the poor, but only to those who seem to be "at ease in Zion." Unlike the gift of the Holy Ghost, which the magician, Simon, sought to purchase of Saint Peter, and for which he received a withering curse. "Thy money perish with thee, because thou hast thought the gift of God may be purchased with money."

Mrs. Eddy's lessons are "Three Hundred Dollars," "Tuition for all strictly in advance." She says, "When God called me to set a price on Christian Science—mind healing, I could think of no financial equivalent for the impartation of knowledge of that Divine Power which heals, but I was led to name three hundred dollars for the price of each pupil in one course of lessons at my college, a starting sum for tuition lasting barely three weeks. This amount troubled me; I shrank from asking it, but was finally led by a strange Providence to accept this fee. God has since shown me in multitudinous ways the wisdom of this action."

How different from the command of Jesus to his disciples, "Heal the sick. Freely ye have received; freely give." It must be indeed a "strange Providence" that directs this avaricious woman to extort from her dupes twenty-five dolars a lesson for teaching them a lot of senseless jargon.

She afterwards cut the course to seven lectures, but retained the God appointed fee of three hundred dollars, or over forty-two dollars per lesson. She further says, "Christian Science demonstrates that the patient who pays whatever he is able to pay for being healed is more apt to recover than he who withholds the slight equivalent for health."

A good many of us would like for our patients to absorb this idea. If they would, our bank accounts would be larger and doctors would not be considered such poor business men.

Although thrice married herself, she disapproves of marriage, and no marriages are ever performed in Christian Science churches, as they are "synonymous with legalized lust." How is it possible for husband and wife who love and respect each other or parents who love and adore their children to have any feeling but of supreme contempt for this woman and her odious teachings? Yet there are some very pleasant, fairly well educated people, who have embraced them, and it is our duty to find the causes that lead to their deception.

The most attractive feature of the Christian Sceince religion is the manner in which they insist upon healthfulness and the necessity for cheerfulness, self-forgetfulness and composure,

Their religion teaches them to forget their own sufferings and distresses and to take an interest in outside things. This is the only grain of truth in the whole thing and is simply suggestive therapeutics which has been adopted by Dr. Worcester in his Immanuel Movement.

It is a reaction from materialism to extreme idealism by persons who are found in every community with a tendency to go to extremes with their ideas. There are always people eager for something new, seeking for miracles, and willing to believe it possible to obtain complete mastery over nature.

What should be our attitude towards them?

1. To show the absurdities of their teaching and the danger in its spread.

2. Insist on their reporting to the health department all births, deaths, and contagious diseases, and that in the latter case they take the proper precautions to protect the community.

3. Secure for small children the protection from Christian Science extravagancies, and not allow the murder of innocents by the neglect of deluded parents.

What is the future of Christian Science? It will pass as other social, religious and medical fallacies have done, such as Dowieism, Perkinism, Theosophy, Thompsonianism, and others that have gone before.

### LITERATURE:

The Bible.

Science and Health with Key to the Scriptures, Eddy.

Miscellaneous Writings, Eddy.

Johns Hopkins Hospital Bulletin No. 111, Marshall.

The Religio-Medical Masquerade, Peabody.

